# NMR-Based Characterization of the Interaction between Yeast Oxa1-CTD and Ribosomes

**DOI:** 10.3390/ijms241914657

**Published:** 2023-09-28

**Authors:** Yong Liu, Jing Yang, Maosen Ruan, Huiqin Zhang, Junfeng Wang, Yunyan Li

**Affiliations:** 1 High Magnetic Field Laboratory, Key Laboratory of High Magnetic Field and Ion Beam Physical Biology, Hefei Institutes of Physical Science, Chinese Academy of Sciences, Hefei 230031, China; yongliu6@mail.ustc.edu.cn (Y.L.); yj91@hmfl.ac.cn (J.Y.); maosen@hmfl.ac.cn (M.R.); zhanghuiqing0619@163.com (H.Z.); 2 Science Island Branch of Graduate School, University of Science and Technology of China, Hefei 230026, China; 3 Institutes of Physical Science and Information Technology, Anhui University, Hefei 230601, China

**Keywords:** Oxa1 insertase, C-terminus, ribosome, interactions, NMR, structural transitions

## Abstract

In mitochondria, the major subunits of oxidative phosphorylation complexes are translated by the mitochondrial ribosome (mito-ribosome). The correct insertion and assembly of these subunits into the inner mitochondrial membrane (IMM) are facilitated by mitochondrial oxidase assembly protein 1 (Oxa1) during the translation process. This co-translational insertion process involves an association between the mito-ribosome and the C-terminus of Oxa1 (Oxa1-CTD) Nuclear magnetic resonance (NMR) methods were mainly used to investigate the structural characterization of yeast Oxa1-CTD and its mode of interaction with the *E. coli* 70S ribosome. Oxa1-CTD forms a transient α-helical structure within the residues P342–Q385, which were reported to form an α-helix when combining with the ribosome. Two conserved contact sites that could interact with the ribosome were further identified. The first site was located on the very end of the N-terminus (V321–I327), and the second one encompassed a stretch of amino acid residues I348–Q370. Based on our discoveries and previous reports, a model has been proposed in which Oxa1-CTD interacts with ribosomes, accompanied by transient-to-stable transitions at the second contact site. These observations may enhance our understanding of the potential role of Oxa1-CTD in facilitating the assembly of oxidative phosphorylation complexes and provide insight into the structural characteristics of Oxa1-CTD.

## 1. Introduction

Eukaryotic cell host translation is processed in three distinct compartments: the cytosol, the mitochondria [1], and the chloroplasts (in photosynthetic cells). The majority of mitochondrial proteins are encoded by nuclear genes and are subsequently imported into the mitochondria following their synthesis in the cytosol. However, a small yet crucial subset of proteins is encoded by the mitochondrial genome. In the yeast *S. cerevisiae*, only eight proteins are synthesized by the mitochondrial ribosome (mito-ribosome). These include the core subunits 1–3 (Cox1, Cox2, and Cox3) of cytochrome *c* oxidase; cytochrome *b* of the cytochrome *bc1* complex; Atp6, Atp8, and Atp9 of the ATPase; and the small ribosome subunit Var1 [2]. These mito-ribosome-encoded proteins are predominantly hydrophobic membrane proteins. During their synthesis, they are inserted into the inner mitochondrial membrane (IMM) with the aid of protein insertion machinery [3] through a process known as co-translational insertion [4].

Mitochondrial oxidase assembly protein 1 (Oxa1), a member of the YidC/Oxa1/Alb3 family [3], plays a critical role in protein insertion and assembly. Within eukaryotes, the YidC/Oxa1/Alb3 family includes two members that span the membrane five times: Oxa1 and Oxa2 (Cox18) in the IMM, alongside Alb3 and Alb4 in the chloroplast thylakoid membrane [5]. Notably, the two paralogs in each system are distinguished by unique traits within their C-terminal regions [5]. Specifically, mitochondrial Oxa1 boasts an extended, positively charged C-terminal region responsible for binding to the mito-ribosome—a feature absent in Oxa2. While Oxa1 operates during co-translational insertion [6,7], Oxa2 functions post-translationally. In the chloroplast, Alb3’s elongated C-terminal region interacts with the specific targeting protein SRP43, in contrast to Alb4, which lacks this domain. Among most Gram-positive bacteria, the YidC1 and YidC2 paralogs span the membrane five times, with YidC2 exhibits an extended C-terminal domain that directly binds to the ribosome—a feature absent in YidC1 [8]. The mentioned family members and the topological structures of YidC/Oxa1/Alb3 are shown in Figure S1. These distinct attributes of the C-terminal regions underscore their crucial roles in ribosomal interactions and the intricate process of protein insertion.

The C-terminal domain of Oxa1 (Oxa1-CTD) plays a critical role in the biogenesis of respiratory chain complexes such as cytochrome *c* oxidase, the cytochrome *bc1* complex, and ATP synthase [4,9,10]. The deletion or truncation of Oxa1-CTD leads to a significant reduction in the activity of respiratory chain complexes and impairs the co-translational insertion and membrane integration of specific subunits [1,4]. The matrix-exposed Oxa1-CTD possesses specific features that are crucial for its insertion activity, including a conserved basic nature and the potential to form a coiled-coil structure [11,12]. The Oxa1-CTD physically interacts with mito-ribosomes and constitutes essential binding sites [4,13].

Despite the projected crucial role of CTD in Oxa1’s function, there are currently no structural data available to support this claim. A detailed characterization of the structure and dynamics of the Oxa1-CTD alone and in complex with ribosomes has been carried out. Through nuclear magnetic resonance (NMR) experiments, it has been demonstrated that Oxa1-CTD performs as an intrinsic random coil conformation, and its interactions with ribosomes on a per-residue level have been mapped.

## 2. Results

### 2.1. Sequence Alignment of Oxa1

**Figure 1:**
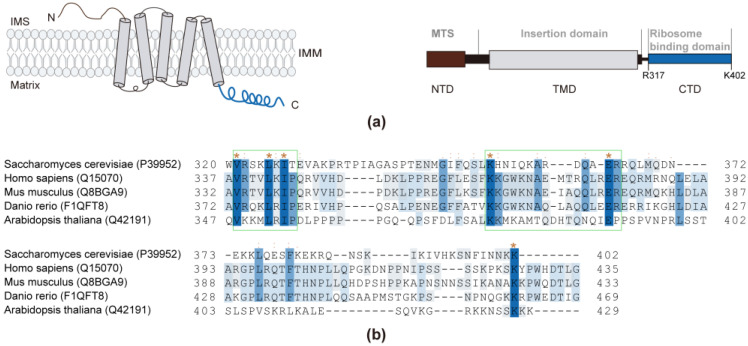
Oxa1 constructs and sequence alignment of Oxa1-CTD. (**a**) Oxa1 comprises the N-terminal domain (NTD), insertion domain (TMD), and C-terminal domain (CTD). The abbreviation MTS represents the mitochondrial targeting sequence. (**b**) Sequence alignment of Oxa1-CTD across the yeast *S. cerevisiae*, *Homo sapiens*, *Mus musculus*, *Danio rerio*, and *Arabidopsis thaliana*. Residues that are invariant in all three sequences are shaded in dark blue and marked with an asterisk (*). Partially conserved and much less conserved residues are shaded in blue and light blue, respectively. The sequence alignment was performed using the Align tool available on UniProt.

Oxa1 consists of three main domains, as depicted in Figure 1a: a N-terminal domain (NTD), containing a mitochondrial target sequence (MST); a membrane-embedded core region comprised of five transmembrane spans (TMD), responsible for catalyzing protein insertion reactions; and a C-terminus (CTD) that extends into the mitochondrial matrix [14]. Analyses of the primary sequence alignment of Oxa1 from yeast *S. cerevisiae*, *Homo sapiens*, *Mus musculus*, *Danio rerio*, and *Arabidopsis thaliana* were conducted. The alignment results illustrate that the membrane-embedded core TMD exhibits a higher sequence identity compared to the MST and CTD (Figure S2). Notably, the very end of the N-terminus (V321–P328) and a motif encompassing a stretch of amino acid residues (K353–R365) within the CTD demonstrated significant homology in conserved hydrophobic and basic residues, such as V321, R322, L325, I327, K353, A359, and R365 (as indicated in Figure 1b). These conserved residues suggest the potential for interactions with the ribosome.

### 2.2. Oxa1-CTD Protein Preparation

**Figure 2:**
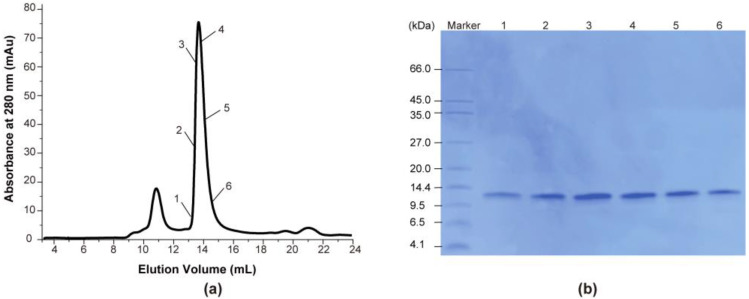
Purification and determination of the oligomeric state of Oxa1-CTD. (**a**) The elution profile of Oxa1-CTD from the Superdex 75 increase 10/300 GL column in NMR buffer (20 mM HEPES pH 7.2, 75 mM NaCl, 20 mM MgCl_2_, 5 mM β-mercaptoenthanol). (**b**) Coomassie blue staining of SDS-PAGE gels were used to evaluate the purity of the target Oxa1-CTD protein sample.

As depicted by the blue curve in Figure 1a, the yeast *S. cerevisiae* Oxa1-CTD studied in this research consists of residues ranging from W320 to K402 (pI = 10.5). The target protein was expressed in *Escherichia coli (E. coli*) supernatant and subsequently purified using nickel affinity chromatography and size exclusion chromatography with a Superdex 75 increase 10/300 GL column. As depicted in Figure 2a, the size exclusion chromatography revealed two symmetrical and sharp peaks, confirming the high protein purity and homogeneity of each peak. Considering the elution volumes of Carbonic anhydrase (~11 mL, 29 kDa) and Ribonuclease A (~14 mL, 13.7 kDa) as stated in the Cytival Manual, it can be inferred that the first peak—appearing at approximately 11 mL—consisted of Oxa1-CTD dimers, while the second peak at ~14 mL contained Oxa1-CTD monomers. The previously characterized cryo-EM structures indicated that Oxa1L exists as a monomer [15]; therefore, the second peak represents our target protein. Additionally, SDS-PAGE analysis (Figure 2b) confirmed the high purity of the target proteins in this peak.

### 2.3. Secondary Structure Analysis of Oxa1-CTD

**Figure 3:**
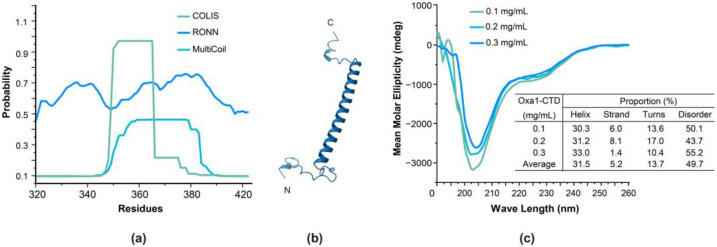
Secondary structural analysis of Oxa1-CTD. (**a**) The coiled-coil prediction of Oxa1-CTD by the COLIS and MultiCoil programs and the disordered prediction of Oxa1-CTD by the RONN program. (**b**) The predicted structure of the Oxa1-CTD by using the AlphaFold2 algorithm. (**c**) CD spectra of Oxa1-CTD at 0.1, 0.2, and 0.3 mg/mL concentrations.

In a previous study, the formation of an α-helical structure in Oxa1-CTD in 50% trifluoroethanol was reported [4]. Here, the secondary structure of Oxa1-CTD was initially predicted using various online programs. As shown in Figure 3a, the COLIS program predicted a probability of over 90% for coiled-coil formation in residues Q350 to R365 [16,17], while MultiCoil predicted no more than a 50% probability for residues N355 to E382 [18]. On the other hand, RONN predicted that the whole region was disordered, with a probability over 50% [19]. Additionally, the three-dimensional structure of Oxa1-CTD predicted by the AlphaFold2 algorithm [20] included a major helical segment spanning residues F349–S387, as well as two other shorter helical segments. Overall, the four prediction results showed some differences, indicating the need for further investigation.

The Circular dichroism (CD) spectra of the Oxa1-CTD at three different concentrations are shown in Figure 3c. Overall, the CD curve of the Oxa1-CTD showed a pattern resembling a random coil, with a negative band of significant magnitude at around 200 nm [21], indicative of a disordered protein. The molar ellipticity ratio [θ_222_/θ_208_] of approximately 0.38 suggests that the Oxa1-CTD did not adopt a coiled-coil motif, which is typically observed with a value of 1.03 for two standard coiled-coil helices [22,23]. However, the spectra displayed two negative bands at around 222 and 208 nm [21], as well as a positive band at around 190 nm, demonstrating the presence of an α-helix in the Oxa1-CTD. The compositions of the secondary structures analyzed and listed in the table inserted in Figure 3c, revealed that Oxa1-CTD consisted of 31.5% α-helix, 5.2% strand, 13.7% turns, and a 49.7% disordered structure, on average. Collectively, the CD results indicated that Oxa1-CTD is a disordered protein with a portion of α-helix. These results are consistent with the prediction from the MultiCoil and RONN programs.

**Figure 4:**
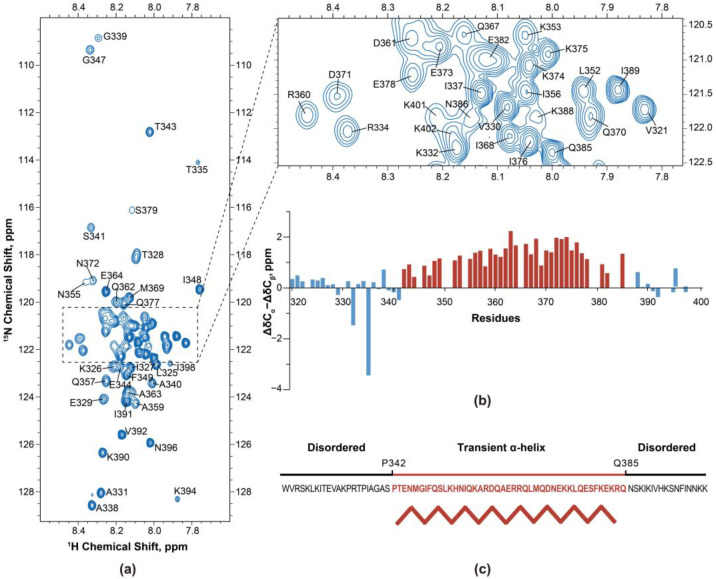
Backbone assignment and secondary structure determination of Oxa1-CTD using nuclear magnetic resonance (NMR) methods. (**a**) Backbone assignment of signals in the 2D ^1^H-^15^N heteronuclear single quantum coherence (HSQC) spectrum was achieved for most residues. However, certain residues such as S323, Q350, S379, N399, and K402 remained unassigned due to either the absence of their resonances in the spectra or significant spin system overlap in both the ^1^H and ^15^N dimensions. (**b**) Secondary chemical shifts of Oxa1-CTD. The regions encompassing residues V321–S341 and K388–I398 were disordered, while the region of residues P342–Q385 formed a transient α-helical structure. (**c**) Overview of Oxa1-CTD secondary structure as suggested by NMR secondary chemical shifts.

Standard NMR experiments, including 3D HNCO, HN(CA)CO, HNCACB, and CBCA(CO)NH were conducted to obtain the backbone assignment of the Oxa1-CTD. Out of 80 non-proline amide proton (^1^H_N_) and nitrogen atom (^15^N) chemical shifts related to wild type residues, 65 (81%) were successfully assigned. The assigned backbone chemical shifts of Oxa1-CTD have been deposited in the BioMagResBank (BMRB accession number 52130). An example of four connected strips from the HNCACB spectrum are shown in Figure S3. Residues that could not be unambiguously assigned due to a lack of resonances in the spectra or intense spin system overlap in both the amide ^1^H_N_ and ^15^N were excluded. The two-dimensional ^1^H-^15^N heteronuclear single quantum coherence (HSQC) spectrum with residue assignment (Figure 4a) showed a narrow chemical shift dispersion in the ^1^H dimension (gathered in the 7.8–8.5 ppm region), characteristic of disordered or helix-forming residues. The resonance positions of glycine, serine, and threonine residues appeared in discrete regions close to their tabulated random coil backbone ^15^N chemical shift, as typically observed for unstructured protein fragments or unfolded polypeptides lacking a regular secondary structure.

The secondary chemical shift, ΔδC_α_ − ΔδC_β_, provides information concerning the protein secondary structure [24,25]. C_α_ atoms tend to have positive Δδ in α-helices but negative Δδ in β-strands, while C_β_ atoms show the opposite behavior. Consecutive values of about 3 ± 1 ppm are typically interpreted as showing a well-defined α-helix structure [26]. Lower values indicate partially formed or transient structures, while values around 0 ppm indicate a random coil conformation [27]. Assessing the secondary structure of Oxa1-CTD, the ΔδC_α_ − ΔδC_β_ values were calculated and are visualized in Figure 4b. Residues V321–S341 displayed values lower than 0.5, indicating disorder. Residues P342 to Q385 showed values of around +1 to +2, indicating a tendency to form a transient α-helix [22]. The C-terminal region (K388 to I398) showed values of around −0.2 to +0.4, suggesting intrinsic disorder. These findings are in line with the CD spectra. Given Oxa1-CTD’s potential for forming a coiled-coil in some regions [4], it is speculated that the transient α-helical region (P342 to Q385) is responsible for the coiled-coil formation.

### 2.4. Oxa1-Ribosome Interactions

**Figure 5:**
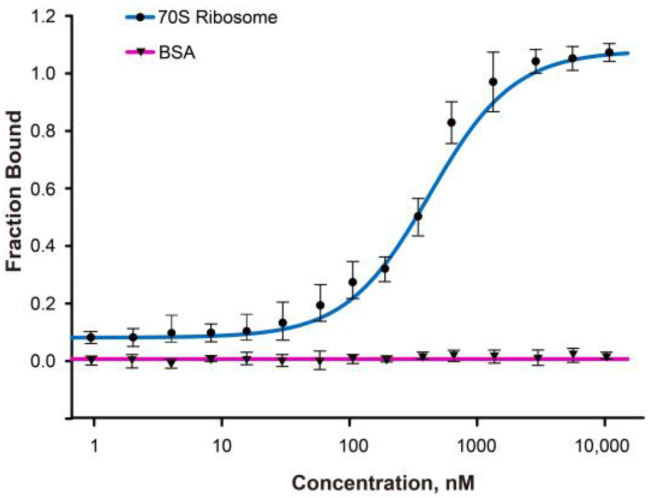
Affinity determination of the interaction between the Oxa1-CTD and the *E. coli* 70S ribosome. Microscale thermophoresis curve of the Oxa1-CTD with 70S (dark blue) and a BSA control (pure magenta).

Microscale thermophoresis (MST) assays were performed to measure the binding affinity between the Oxa1-CTD and *E. coli* 70S ribosome. The analysis (Figure 5) showed that the Oxa1-CTD has a binding affinity of 0.45 μM towards the ribosome, while it showed negligible binding to BSA, confirming the specificity of the interaction between the Oxa1-CTD and the ribosome.

NMR titration experiments were performed to study the binding of the Oxa1-CTD to the ribosome at the residue level. A series of ^1^H-^15^N HSQC spectra were recorded utilizing ^15^N-labelled Oxa1-CTD with increasing amounts of unlabelled *E. coli* 70S ribosome to observe any changes in the peak intensities. The interaction between the Oxa1-CTD and ribosome could lead to slower tumbling of the binding residues, causing broadened peaks and decreased peak intensity [23].

**Figure 6:**
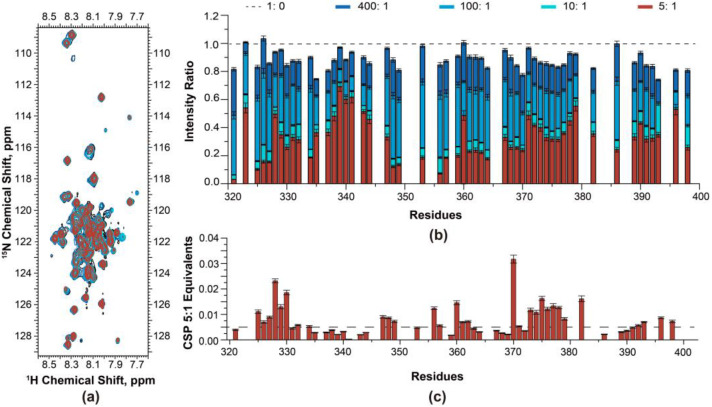
Analyses of Oxa1-CTD and ribosome interactions. (**a**) 2D ^1^H-^15^N HSQC spectra of ^15^N-labelled Oxa1-CTD titrated with *Escherichia coli* (*E. coli*) 70S ribosome. ^15^N-labelled Oxa1-CTD: *E. coli* 70S ribosome ratios: 400:1 (dark blue), 100:1 (blue), 10:1 (cyan), and 5:1 (red). The reference 2D ^1^H-^15^N HSQC spectrum of ^15^N-labelled Oxa1-CTD without ribosome is shown in black. (**b**) Relative peak intensities (intensity ratio) of ^15^N-labelled Oxa1-CTD backbone amide resonances during NMR titration (normalized to ^15^N-labelled Oxa1-CTD without ribosome, black dashed line, value = 1) (**c**) Chemical shift perturbations observed in the 2D ^1^H-^15^N HSQC spectra at a 5:1 ^15^N-labelled Oxa1-CTD to ribosome ratio compared to spectra without ribosome. The horizontal dashed line indicates a threshold of 0.005 ppm.

In the initial molar ration of 400:1 of Oxa1-CTD to ribosome, NMR peak intensities (dark blue circles and bars in Figure 6a,b) showed a slight decrease compared to the reference without the ribosome. As the ratio decreased to 100:1, the peak intensities of V321 and L325 decreased noticeably (blue circles and bars in Figure 6a,b). At a ratio of 10:1, the peak intensities of the residues V321–I327 and I348–Q370 decreased by approximately 80%, while the rest of the segments showed a signal loss of around 50% (cyan circles and bars in Figure 6a,b). These two segments (V321–I327 and I348–Q370) further experienced an intensity decrease at a ratio of 5:1 (red circles and bars in Figure 6a,b).

Chemical shift perturbations (CSPs) in NMR are a valuable method for studying protein–ligand interactions, protein–protein interactions, and conformational changes in proteins. CSPs were also measured for the backbone ^1^H_N_ and ^15^N of the Oxa1-CTD at a ratio of 5:1. Figure 6c shows clear chemical shift changes in residues K328–V330, Q370, E373–E378, and E382, which correspond to the segments with a significant intensity decrease observed in the NMR titration. On the other hand, CSPs are much smaller in residues K388 to I398 at the C-terminal end, despite their considerable peak intensity decrease. The NMR titration results explicitly indicated that the very end of the N-terminus V321–I327 and residues I348–Q370 of the Oxa1-CTD were primarily responsible for binding to the ribosome.

## 3. Discussion

Oxidative phosphorylation (OXPHOS) is a vital process in mitochondria that produces ATP, serving as the main energy source for cells. The interaction between Oxa1-CTD and the mito-ribosome plays a crucial role in the accurate assembly of OXPHOS complexes. This study specifically focused on soluble Oxa1-CTD, with the aim of determining its structure and its interactions with the ribosome.

Oxa1-CTD was characterized as an intrinsically disordered protein (IDP) with no stable secondary structure. Our approach involved prediction, circular dichroism (CD), and nuclear magnetic resonance (NMR). The analysis revealed that Oxa1-CTD is an IDP, with a specific region comprising residues P342–Q385 exhibiting a transient α-helical structure. It is important to note that the functions of IDPs are diverse and context-dependent, and their roles can vary across different biological systems and processes [28,29]. For example, some IDPs are involved in cellular signaling pathways and regulatory networks, acting as molecular switches that undergo disorder-to-order transitions upon binding to specific partners or post-translational modifications [30]. In the case of Oxa1-CTD, although it did not exhibit stable secondary structures, it could form transient secondary structure elements, which could be further stabilized through binding to other partners.

Oxa1 functions as an insertase in the IMM by interacting with the matrix side of the Oxa1-CTD and the large ribosomal subunit. In order to explore the dynamic interactions between Oxa1-CTD and the *E. coli* 70S ribosome, MST experiments were conducted. The use of the *E. coli* 70S ribosome as an alternative to yeast ribosomes presents several advantages, such as easy purification and high stability and activity, eliminating the need for complex purification processes. While the *E. coli* 70S ribosome cannot entirely substitute for the yeast ribosome, it maintains a similar interaction mode with *E. coli* 70S. Through MST analysis, we identified binding affinities—likely falling within the micromolar (μM) range—indicative of a moderate to relatively strong interaction. This level of affinity likely plays a significant role in the precise control of the co-translational insertion process, ensuring the accurate synthesis and assembly of nascent chains.

A detailed analysis of peak intensities and chemical shift perturbations from the NMR titration experiments revealed two main regions of the Oxa1-CTD that specifically interact with the ribosome. The first region is located at the very end of the N-terminus of the Oxa1-CTD (V321–I327), with the residues V321, R322, L325, and I327 conserved across yeast *S. cerevisiae*, *Homo sapiens*, *Mus musculus*, *Danio rerio*, and *Arabidopsis thaliana*, indicating a conserved contact site. The second region is situated within residues I348–Q370, featuring the conserved residues K353, A359, and R365, suggesting another conserved contact site.

Our identification of two key contact regions between the Oxa1-CTD and the ribosome holds promise for providing new insights into previously unresolved interaction sites. In a recent breakthrough, the cryo-EM structure of human mitochondrial ribosome bound to Oxa1L revealed the existence of three distinct interaction sites between the human mitochondrial ribosome and the C-terminus of Oxa1L: Site 1 is located at the very end of the C-terminus of human Oxa1L, encompassing residues Y428 to G435—a feature absent in yeast (refer to Figure 1b). Due to limitations in resolution, the specific amino acids at Sites 2 and 3 remain undetermined. However, the two contact regions we have identified hold the potential to help bridge the knowledge gaps concerning Site 2 and 3 interactions between the mitochondrial ribosome and human Oxa1L [15]. Previous suggestions have proposed that Site 2 is 27 Å away from Site 1, spanning approximately 40 residues. The second binding site, I348–Q370, corresponding to human residues F361–R386, indicates an exact 40 amino acid separation from Y428. Most notably, the Oxa1-CTD residue A359 aligns with human Oxa1L residue A372, which has been shown biochemically to be a key contact point with the ribosome [31]. These demonstrate a strong preservation of the ribosome-binding interface we characterized in yeast, supporting that it is responsible for Site 2 in the human complex. Likewise, the first contact site at the Oxa1-CTD’s N-terminus also shares several conserved residues when compared to human Oxa1L, implying a comparable binding function to Site 3.

**Figure 7:**
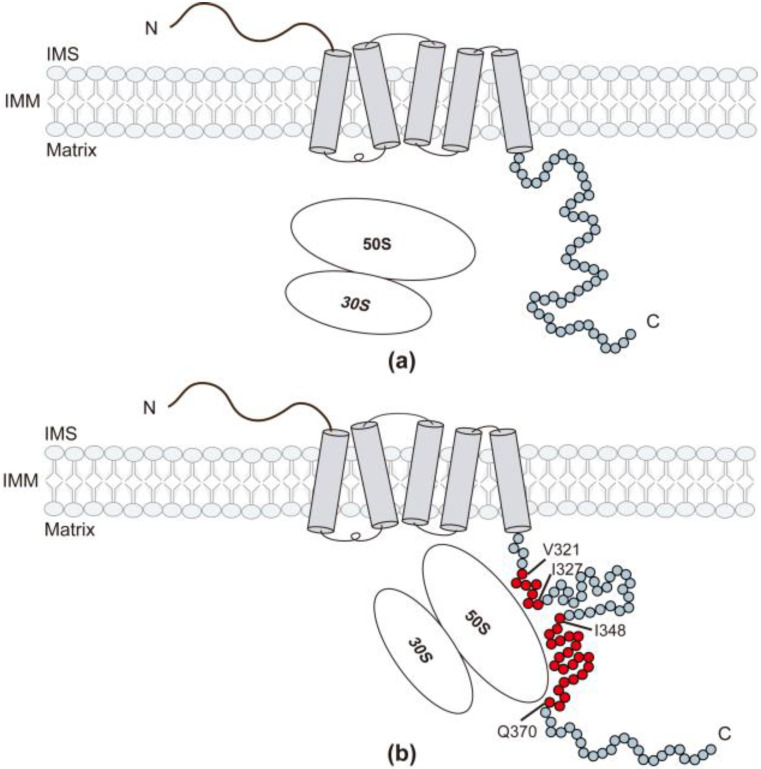
Altered conformation for the interaction between Oxa1-CTD and the ribosome. (**a**) The Oxa1-CTD is intrinsically disordered, with a transient α-helical structure (residues P342–Q385) prior to ribosome binding. (**b**) Two main regions of Oxa1-CTD specifically interacting with the ribosome: residues V321–I327 and I348–Q370, which later undergo transient-to-stable transitions. Amino acids within Oxa1-CTD are depicted as circles. Those amino acids involved in ribosome interactions are marked in red, while the remaining amino acids are depicted in light blue.

Drawing from the published α-helical structure of the Oxa1-CTD in a ribosome-Oxa1 cryo-EM map [15,32], along with the obtained data, a model is proposed: Initially, Oxa1-CTD exists as intrinsically disordered, with a transient α-helical structure spanning residues P342–Q385 (Figure 7a). During the synthesis of nascent chains, the mito-ribosome interacts with Oxa1-CTD, resulting in transient-to-stable transitions and leading to the adoption of an α-helical structure (Figure 7b). This dynamic process is proposed to assist in the correct insertion of the nascent chain into the IMM.

The tight association between the mito-ribosome and Oxa1 during the co-translational insertion process offers several advantages: (1) it enables efficient protein insertion by ensuring the prompt delivery of nascent polypeptides to Oxa1 for insertion into the mitochondrial inner membrane; (2) the interaction assists in protein folding, guiding and stabilizing the folding process during membrane insertion; (3) the coordinated assembly of multi-subunit complexes is made possible, ensuring the correct stoichiometry and structural integrity of these complexes; (4) the interaction serves as a quality control mechanism, preventing the misfolding and aggregation of nascent polypeptides by allowing prompt recognition and targeting for degradation or refolding. These advantages are crucial for the proper functioning of mitochondrial proteins and overall mitochondrial function.

## 4. Materials and Methods

### 4.1. Cloning, Expression, and Purification

Genes encoding yeast *S. cerevisiae* Oxa1 (NCBI reference sequence: NM_001179044) were cloned by Polymerase Chain Reaction into the pET-14b vector (Novagen, Darmstadt, Germany) with a N-terminal 6× His-tag and a thrombin cleavage site, using the yeast *Saccharomyces cerevisiae* cDNA library as a template. The following primers were used in the PCR: (a) 5′-CTGACATATGTGGGTTCGTTCGA-3′ (NdeI restriction site, Takara Bio USA, San Jose, CA, USA) and (b) 5′-TACCGGATCCTTATTTTTGTTA (BamHI restriction site, Takara Bio USA, CA, USA) for Oxa1-CTD. The plasmid pET14b-Oxa1-CTD (W320-K402) was expressed in *E. coli* BL21(DE3) cells ((Novagen, Darmstadt, Germany)). Overexpression of Oxa1-CTD was induced with 1 mM isopropyl β-D-thiogalatopyranoside (IPTG, Sigma-Aldrich, MO, USA) when the optical density at 600 nm (OD_600_) reached 0.6, performed at 37 °C for 4 h in either Luria-Bertani (LB) liquid media or M9 medium. In the M9 medium, ^15^NH_4_Cl (1 g/L) and/or ^13^C_6_ glucose (4 g/L) were used as the sole nitrogen and carbon source for ^15^N- or ^15^N/^13^C-labelled protein, depending on the desired labelling scheme: ^15^N-labelled, ^15^N/^13^C-labelled; the ^15^NH_4_Cl (1 g/L) and ^13^C_6_ glucose were purchased from Cambridge Isotope Laboratories, Inc., Tewksbury, MA, USA.

Cells were resuspended in lysis buffer (50 mM HEPES pH 7.2, 150 mM NaCl) and disrupted at 4 °C using a high-pressure homogenizer (Union-Biotech (Shanghai), Shanghai, China) at 850 psi and then centrifuged at 16,000× *g* for 30 min. The supernatant was filtered through a 0.45 µm filter and then subjected to nickel affinity chromatography (Ni Sepharose™ 6 Fast Flow, GE Healthcare, GE Healthcare, Piscataway, NJ, USA) and eluted by a gradient to 500 mM imidazole in the same buffer. After this, the protein was further purified by size exclusion chromatography (SEC, GE Healthcare, Piscataway, NJ, USA) using an ÄKTA FPLC system with the Superdex 75 increase column (Cytiva, Marlborough, MA, USA), and the purity of the protein was analyzed by SDS-PAGE. Finally, the protein was concentrated for the NMR experiments.

### 4.2. Isolation of E. coli Ribosome

The ribosomes were extracted from *E. coli* MRE600 cells (American Type Culture Collection, VA, USA) using a Ribosome isolation kit (Solarbio Science & Technology, Beijing, China): 40 g of wet cells were resuspended in 50 mL resuspension buffer (20 mM HEPES-KOH pH 7.6, 6 mM MgCl_2_, 30 mM NH_4_Cl and 4 mM β-mercaptoethanol) and disrupted at 4 °C using a homogenizer at 850 psi and then centrifuged at 16,000× *g* for 10 min. The supernatant was centrifuged at 20,000× *g* and 4 °C for 10 min. After this, the supernatant was collected and centrifuged again at 100,000–120,000× *g* and 4 °C for 60 min. Finally, the precipitate was collected, added to 6 mL cold buffer (10 mM HEPES-KOH pH 7.6, 1 M NH_4_Cl, 40 mM MgCl_2_, 6 mM β-mercaptoethanol), mixed well, and then centrifuged at 100,000–120,000× *g* and 4 °C for 60 min. The precipitate was collected as 70S ribosomes.

### 4.3. Circular Dichroism (CD) Spectroscopy

The far-UV CD spectra of 0.1, 0.2 and 0.3 mg/mL Oxa1-CTD in 10 mM phosphate buffer (pH 7.2) were measured at 20 °C using a Jasco J-1700 Circular Dichroism Spectrophotometer with the settings: 260 nm to 190 nm, 1 nm steps, 1 s integration time for each wavelength. Three successive scans were performed for each concentration. Spectra were the average of three scans. Data was processed with Spectra Analysis software (JASCO, Spectra Manager 2.0). Background was subtracted prior to deconvolution.

### 4.4. NMR Sample Preparation, Data Collection, and Assignment

NMR samples were prepared in an NMR buffer of 20 mM HEPES pH 7.2, 75 mM NaCl, 20 mM MgCl_2_, and 5 mM β-mercaptoethanol, with a protein concentration of 0.5 mM for assignment. All NMR experiments were performed at 298 K on Bruker spectrometers operating at a ^1^H frequency of 850 MHz or 600 MHz, equipped with 5 mm TCI cryogenic probes. Sequence-specific assignments of backbone ^1^H_N_, ^15^N, ^13^C_a_, ^13^C_b_ and ^13^C resonances were accomplished using 3D HNCACB, HN(CO)CACB, HNCO, HN(CA)CO, HNCA, HN(CO)CA, as well as ^1^H-^15^N heteronuclear single quantum coherence (HSQC) experiments. All NMR data were processed with Bruker Topspin 3.6.2 and analyzed by CcpNmr 2.4.

### 4.5. Microscale Thermophoresis (MST) Assays

Oxa1-CTD was labelled with the red fluorescent dye using the Monolith NT^TM^ protein labeling kit, according to the supplied protocol. Binding experiments were performed using the Monolith NT Label-Free system (NanoTemper Technologies, Munich, Germany); in brief, a 20 µM Oxa1-CTD sample was prepared, and its interaction with increasing concentrations of *E. coli* 70S was examined. The concentrations of ribosomal subunits and BSA ranged between 1.21, 2.43, 4,87, 9.75, 19.5, 39, 78, 156, 312, 625, 1250, 2500, 5000, 10,000, and 20,000 nM. The mixture (18 µL) was loaded into a silicon capillary (K002 Monolith NT.115), followed by thermophoresis at 280 nm. All experiments were carried out in a buffer composed of 20 mM HEPES pH 7.2, 75 mM NaCl, and 20 mM MgCl_2_. Each measurement was repeated three times for accuracy. Data analyses were conducted employing MO.Affinity Analysis software (NanoTemper, version 2.3). The equilibrium dissociation constant (*K_d_*) was determined following NanoTemper technologies’ prescribed protocol. A dose–response curve was generated by plotting $1-\frac{F_{i}}{F_{norm}}$ against the ligand concentration. The binding affinity values characterizing the interactions between a protein and its substrate were calculated using the saturation binding curve at equilibrium. The fitting function [33] employed in this calculation was:

$$FB=\frac{\left[AB\right]}{\left[B\right]}=\frac{\left[A\right]+\left[B\right]+K_{d}-\sqrt{{\left(\left[A\right]+\left[B\right]+K_{d}\right)}^{2}-4[AB]}}{2[B]}=1-\frac{F_{i}}{F_{norm}}$$

$\left[A\right]$ concentration of titrated partner *A* (*E. coli* ribosome); $\left[B\right]$: concentration of ligand *B* (Oxa1-CTD); [AB]: concentration of bound complex of *A* and *B*. $K_{d}$ is the equilibrium dissociation constant. The fluorescence of Oxa1-CTD in the absence of the *E. coli* ribosome (defined as $F_{norm}$) and in the presence of the *E. coli* ribosome ($F_{i}$) was recorded.

### 4.6. NMR Titration Assays

NMR titration experiments with unlabelled vacant *E. coli* 70S ribosome were performed using a 45 µM ^15^N labelled Oxa1-CTD sample in the buffer conditions given above. The molar ratios of Oxa1-CTD to 70S for titration were 400:1, 100:1, 10:1, and 5:1. A 2D SOFAST HMQC spectrum was acquired at each concentration. Signal intensity ratios were calculated as bound peak intensity divided by free peak intensity, where bound and free correspond to sample in the presence and absence of 70S ribosome, respectively.

## Data Availability

The data are contained within the article and Supplementary Materials. The original SDS-page pictures were submitted with this article.

## Supplementary Materials

The following supporting information can be downloaded at: https://www.mdpi.com/article/10.3390/ijms241914657/s1.
