# Supplementary material for: NMR-Based Characterization of the Interaction between Yeast Oxa1-CTD and Ribosomes
Source: Int J Mol Sci. 2023 Sep 28;24(19):14657. doi: 10.3390/ijms241914657 (PMC10572626; doi:10.3390/ijms241914657)
Supplement: Supplementary file 1 [file ijms-24-14657-s001.zip › ijms-2603957-supplementary.pdf]

Supplementary Materials for

NMR-based Characterization of the Interaction between Yeast Oxa1-CTD and Ribosome

Authors:

Yong Liu [1,2], Jing Yang [1], Maosen Ruan [1], Huiqin Zhang [1,3], Junfeng wang[1,2,3,*], Yunyan Li [1,*]

This file includes:

Figure S1 to Figure S3

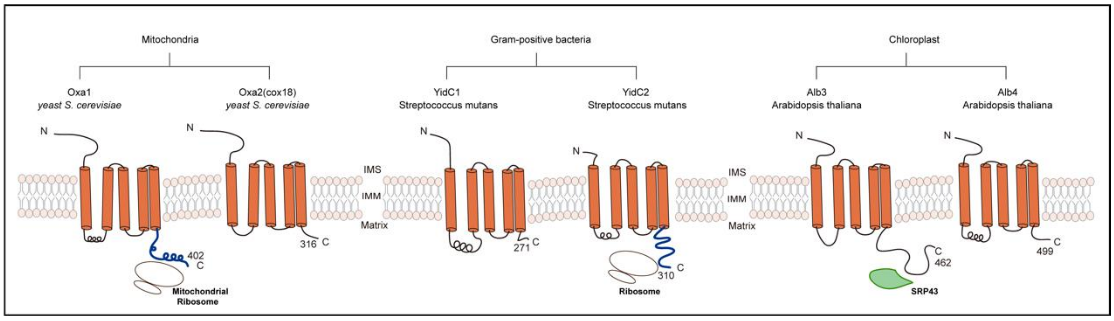

Figure S1. Structural and functional schematic of certain members within the YidC/Oxa1/Alb3 family.

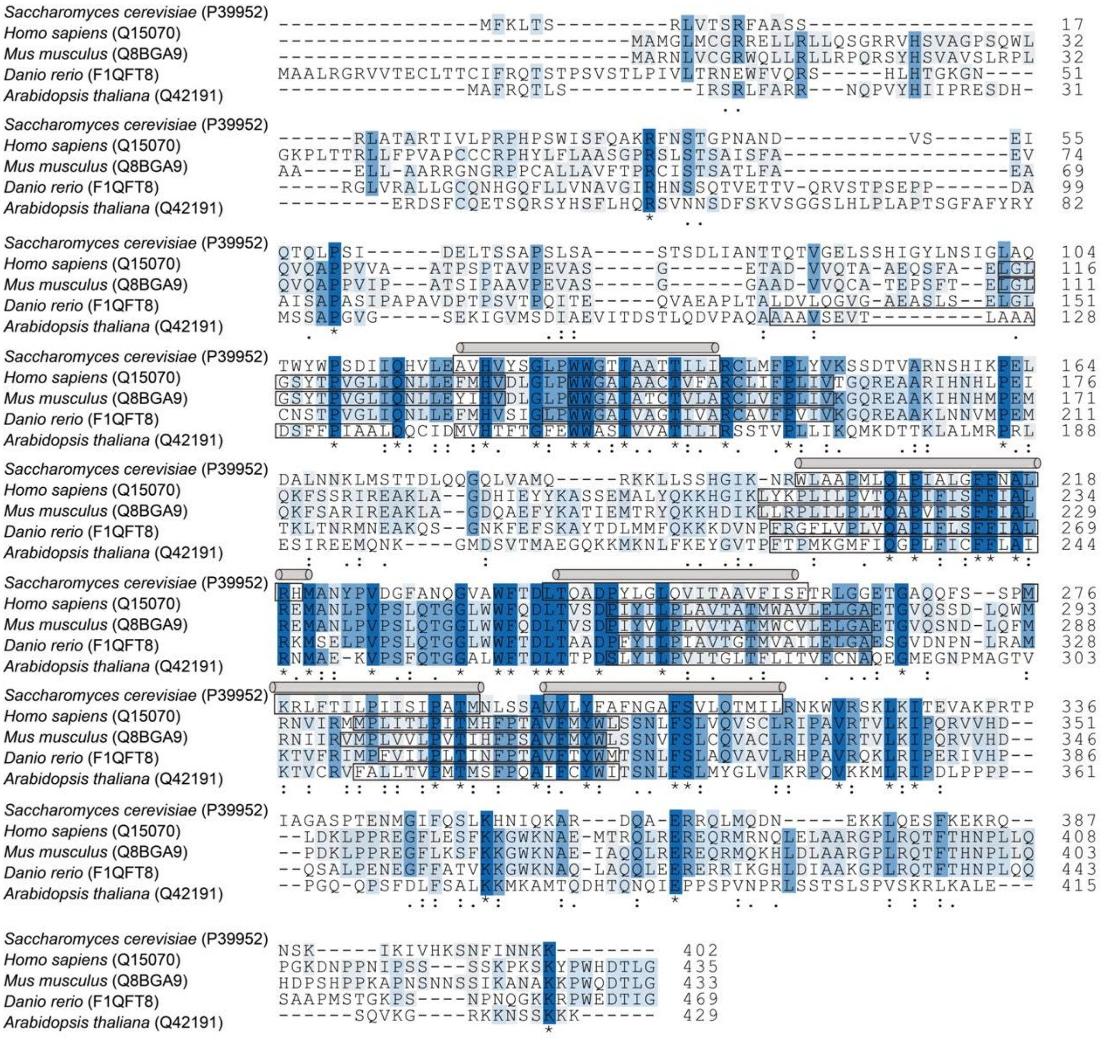

Figure S2. Sequence alignment of full-length Oxa1 across yeast *S. cerevisiae*, *Homo sapiens*, *Mus musculus*, *Danio rerio*, and *Arabidopsis thaliana*. The sequence alignment was performed using the Align tool available on UniProt.

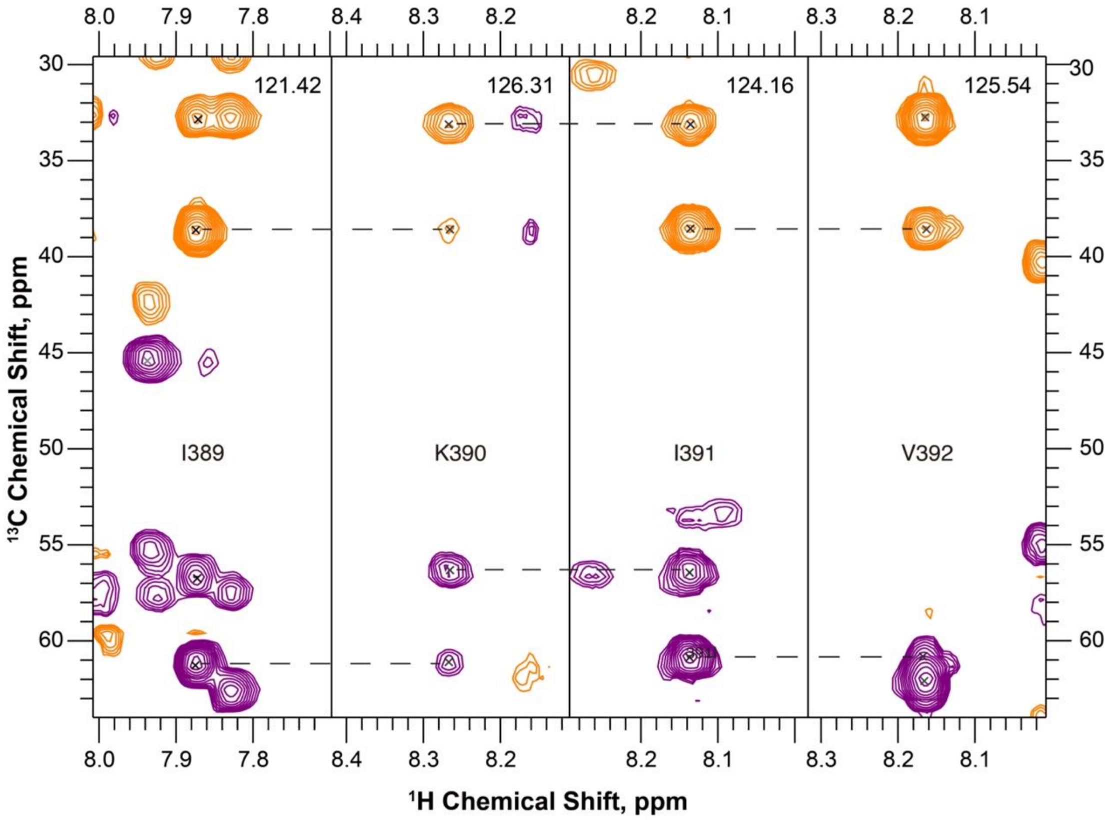

Figure S3. Residue-specific strips (I389-V392) from 3D HNCACB spectrum recorded on 850 MHz spectrometer.
